# Faces of a Changing Climate: Semi-Quantitative Multi-Mycotoxin Analysis of Grain Grown in Exceptional Climatic Conditions in Norway

**DOI:** 10.3390/toxins5101682

**Published:** 2013-09-27

**Authors:** Silvio Uhlig, Gunnar Sundstøl Eriksen, Ingerd Skow Hofgaard, Rudolf Krska, Eduardo Beltrán, Michael Sulyok

**Affiliations:** 1 Section for Chemistry and Toxicology, Norwegian Veterinary Institute, Ullevålsveien 68, Oslo N-0454, Norway; E-Mail: gunnar.eriksen@vetinst.no; 2 Bioforsk Plant Health and Plant Protection, Høgskoleveien 7, Ås N-1430, Norway; E-Mail: ingerd.hofgaard@bioforsk.no; 3 Center for Analytical Chemistry, Department IFA-Tulln, University of Natural Resources and Life Sciences (BOKU), Vienna, Konrad Lorenz Str. 20, Tulln A-3430, Austria; E-Mails: rudolf.krska@boku.ac.at (R.K.); michael.sulyok@boku.ac.at (M.S.); 4 Research Institute for Pesticides and Water, University Jaume I., Castellón de la Plana E-12071, Spain; E-Mail: ebeltran@uji.es

**Keywords:** climate change, fungi, LC-MS, multiplexing, mycotoxin

## Abstract

Recent climatological research predicts a significantly wetter climate in Southern Norway as a result of global warming. Thus, the country has already experienced unusually wet summer seasons in the last three years (2010–2012). The aim of this pilot study was to apply an existing multi-analyte LC-MS/MS method for the semi-quantitative determination of 320 fungal and bacterial metabolites in Norwegian cereal grain samples from the 2011 growing season. Such knowledge could provide important information for future survey and research programmes in Norway. The method includes all regulated and well-known mycotoxins such as aflatoxins, trichothecenes, ochratoxin A, fumonisins and zearalenone. In addition, a wide range of less studied compounds are included in the method, e.g., *Alternaria* toxins, ergot alkaloids and other metabolites produced by fungal species within *Fusarium*, *Penicillium* and *Aspergillus*. Altogether, 46 metabolites, all of fungal origin, were detected in the 76 barley, oats and wheat samples. The analyses confirmed the high prevalence and relatively high concentrations of type-A and -B trichothecenes (e.g., deoxynivalenol up to 7230 µg/kg, HT-2 toxin up to 333 µg/kg). Zearalenone was also among the major mycotoxins detected (maximum concentration 1670 µg/kg). Notably, several other *Fusarium* metabolites such as culmorin, 2-amino-14,16-dimethyloctadecan-3-ol and avenacein Y were co-occurring. Furthermore, the most prevalent *Alternaria* toxin was alternariol with a maximum concentration of 449 µg/kg. A number of *Penicillium* and *Aspergillus* metabolites were also detected in the samples, e.g., sterigmatocystin in concentrations up to 20 µg/kg.

## 1. Introduction

The 2011 weather conditions in Norway can be described as rather extreme both with regard to temperature and precipitation. Observations indicated an average temperature of 1.8 °C more than what is regarded as usual. Consequently, the temperature reached the same level as in 1990 and 2006, which were the warmest years since the recording of climate data started in 1900. At the same time, 2011 was registered as the wettest year since 1900 with a 130% precipitation rate compared to what is regarded as “normal” for the entire country. Notably, the annual precipitation rate in certain areas of Central Norway was as high as 175% of what is regarded as “normal”. Furthermore, the precipitation in June and July, which is especially important for fungal infections during flowering, was reported as 170% and 135% of the expected precipitation for the entire country, respectively. A complete climatological report is available on the homepage of the Meteorological Institute of Norway [1]. Likewise, the summer of the two preceding years were unusually wet in the southern part of the country as well. Hence, it came as no surprise when our national survey programme revealed constantly increasing trichothecene concentrations in grain during the last decade [2]. In this same period, a rise in *Fusarium* infested seeds has been reported [3]. However, as fungi may produce a variety of different metabolites, animals and humans that live on a grain-based diet are exposed to a complex chemical cocktail. Therefore, the composition of this cocktail, rather than certain groups of toxins/metabolites alone, needs to be considered when assessing the safety of grain or grain-based products. Knowledge regarding the commonly co-occurring metabolites in grain can further provide the basis for future toxicological studies on combined effects.

State-of-the-art LC-MS instrumentation is sophisticated and allows for simultaneous analysis of a high number of chemically different molecules. Over the last decade, a LC-MS/MS based method has been developed at the IFA-Tulln that presently includes 320 metabolites of primarily fungal origin [4,5,6,7]. The method has been validated for certain commodities and has successfully been employed for the analysis of a variety of raw and finished food and feed products as well as settled dust [4,5,6,7]. The aim of this pilot study was to apply this multi-analyte method to field grain samples from the main cereal growing districts in Norway from a “worst-case season”, in order to point out which metabolites and metabolite mixtures should be prioritised in the future national survey and research programmes in Norway.

## 2. Results and Discussion

### 2.1. Methodological Considerations

Multiplexing of a wide range of metabolites provides indirect information on the microorganisms that infected the sample commodity and thus gives the analyst an idea of which related compounds it might be worthwhile to look for. The objective of this pilot study was to semi-quantitatively determine the co-occurrence of fungal metabolites in Norwegian grain. Thus, the instrument was calibrated using external calibration, and the investigation of matrix effects was not an objective of this work. Likewise, spike-recovery trials were not included in this study. However, method performance characteristics were determined for certain commodities in previous studies, and the method has successfully been employed for the analysis of a variety of raw and finished food and feed products as well as settled dust [4,5,6,7]. These reports show that the rate of recovery from similar matrices for most metabolites is >60%. The matrix effect in terms of signal suppression/enhancement for grain matrices is generally reported in the range of 70%–130% [4,5,6,7]. Within the scope of this study, the abovementioned limitations were regarded as acceptable, and the reported concentrations must be seen in the light of these methodological limitations.

### 2.2. Metabolites Related to Fusarium

**Figure 1:**
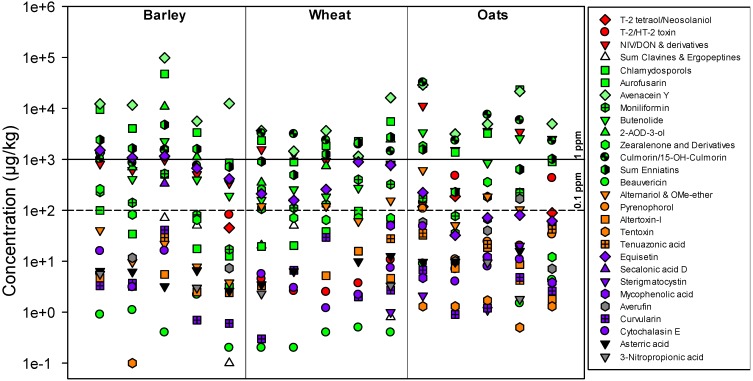
.Scatter plots visualising the co-occurrence of (groups of) fungal metabolites in the five highest contaminated samples of barley, wheat and oats. Colours represent: trichothecenes, red; *Fusarium* metabolites other than trichothecenes, green; *Alternaria* metabolites, orange; *Penicillium*/*Aspergillus* metabolites, purple; ergot alkaloids, white; other fungal metabolites, grey.

Of the 320 metabolites analysed for in this study, a total of 46 fungal metabolites were detected in the grain samples (Table 1, Figure 1). These reflect the commonly observed infection of Norwegian grain with certain *Fusarium* species, especially *F. avenaceum* [8,9]. *F. avenaceum* is likely responsible for the observed contamination with moniliformin (MON), enniatins (ENNs), avenacein Y and 2-amino-14,16-dimethyloctadecan-3-ol (2-AOD-3-ol). Furthermore, the high occurrence of deoxynivalenol (DON), especially in oats, supports previous findings obtained in our national survey programme showing that the contamination of grain with this major trichothecene has constantly increased in recent years [2]. This increase is most likely related to the observed augmented occurrence of *F. graminearum* in Northern Europe as well as Norway and replacement of *F. culmorum* [10,11,12]. Median DON concentrations were 150, 1070, and 383 µg/kg in barley, oats and wheat, respectively, while maximum concentrations were five to seven times higher (Table 1). The detected median concentrations of DON in wheat are generally comparable to what is commonly found in this grain species on a worldwide basis, while the maximum concentration was still well below the most extreme cases reported elsewhere [13]. Median concentrations of DON in oats, found in this study, were about twice as high as reported earlier for Northwestern Europe while the maximum concentrations were comparable [14]. While the metabolite profile of *F. culmorum* is similar to that of *F. graminearum*, the latter appears to be a more potent DON producer. Besides, the amount of *F. graminearum* DNA, but not *F. culmorum* DNA, has been shown to correlate with DON content in grain where both species co-occurred [15]. Only 3-acetyl-DON was detected in the samples, which is interesting with regard to the fact that the 15-acetyl-DON genotype of *F. graminearum* has recently been isolated from Norwegian grain samples. 

The concentrations of glucosylated DON were generally in the range 4%–60% of the unconjugated mycotoxin, which is in accordance with previous reports [2]. However, in two samples from the present study, the relative proportion of DON-3-glucoside/DON was as high as 91% and 153%. Furthermore, the concentration of DON was positively correlated to the concentration of DON-3-glucoside with *R*^2^ coefficients between 0.69 (wheat) and 0.92 (oats). This indicates a relatively constant proportion of the toxin being conjugated *in planta* in the present set of samples. However, we analysed not for other glycosylated trichothecenes due to the current lack of reference material. Importantly, the contamination of oats with DON was significantly higher than that of wheat and barley (Student’s *t*, *p*<0.0001). In addition, the highest amounts of type-A trichothecenes were also detected in oats (Table 1, Figure 1). While T-2 toxin, HT-2 toxin and T-2 tetraol were co-occurring in most oats samples, HT-2 toxin was the dominating analogue. It is today generally accepted that *F. langsethiae* is the species that is primarily responsible for the sometimes substantial contamination of Norwegian oats with type-A trichothecenes [8]. Earlier studies have shown that the contamination of grain from Northern Europe with type-A trichothecenes is often higher than that of grain from Central and Southern Europe [13,14]. Despite this, the median concentrations of T-2 and HT-2 toxin in this study (20.1 and 92.8 µg/kg of T-2 and HT-2 toxin in oats, respectively) were well below commonly reported median concentrations for the toxins in grain [13,14].

**Table 1:** List of detected mycotoxins/fungal metabolites, their prevalence, median of positives and maximum detected amount.

		**Barley (*n* = 20)**			**Oats (*n* = 28)**			**Wheat (*n* = 28)**		
	**Metabolite**	**Positive (%)**	**Median (µg/kg)**	**Maximum (µg/kg)**	**Positive (%)**	**Median (µg/kg)**	**Maximum (µg/kg)**	**Positive (%)**	**Median (µg/kg)**	**Maximum (µg/kg)**
Type-A trichothecenes	T-2 tetraol	30	12.9	43.1	79	54.7	174	0	-	-
	T-2 toxin	30	5.1	14.1	96	20.1	143	79	3.4	5.3
	HT-2 toxin	25	14.2	67.6	82	92.8	333	4	6.1	6.1
	Neosolaniol	5	2.2	2.2	46	3.4	13.8	0	-	-
Type-B trichothecenes	Nivalenol	55	2.9	13.6	93	5.2	45.5	14	2.2	2.4
	Deoxynivalenol	100	150	636	100	1070	7230	100	383	1400
	DON-3-glucoside	100	67.8	270	100	252	2580	100	56.4	152
	3-Acetyl-DON	60	17.8	141	100	128	1380	68	14.0	49.5
Depsipeptides	Enniatin A	100	17.1	185	100	3.7	30.0	100	4.1	92.7
	Enniatin A1	100	145	1,180	100	21.4	263	100	48.0	276
	Enniatin B	100	440	807	100	69.6	662	100	347	874
	Enniatin B1	100	529	2,820	100	65.5	706	100	296	1400
	Enniatin B2	100	23.9	133	100	3.2	24.5	100	15.1	79.3
	Enniatin B3	100	0.2	1.0	100	0.05	0.21	100	0.16	0.62
	Beauvericin	100	0.4	2.2	100	3.5	15.1	100	0.4	1.1
Zearalenone and related compounds	Zearalenone	95	11.4	1340	93	89.9	1670	96	27.5	210
	β-Zearalenol	35	6.8	158	79	7.2	97.2	36	5.4	18.2
	ZEN-4-sulphate	55	3.1	76.7	93	2.6	43.5	82	1.1	18.2
Other *Fusarium* metabolites	Chlamydosporols	75	26.1	509	61	33.7	225	86	16.7	96.2
	Aurofusarin	100	1090	47,300	100	1,350	30,500	100	934	5510
	Avenacein Y	90	6030	98,100	100	4630	28,800	100	1520	16,200
	Moniliformin	100	86.0	522	100	57.2	220	100	88.4	400
	Butenolide	100	231	2260	97	154	3370	86	198	818
	Culmorin	95	292	1440	100	2000	31,500	100	986	3160
	15-Hydroxy-culmorin	40	49.5	70.9	71	117	924	36	70.6	105
	2-AOD-3-ol	35	1100	10,800	0	-	-	36	532	2460
	Equisetin	100	433	2,470	100	56.3	311	100	204	890
		**Barley**			**Oats**			**Wheat**		
	**Metabolite**	**Positive (%)**	**Median (µg/kg)**	**Maximum (µg/kg)**	**Positive (%)**	**Median (µg/kg)**	**Maximum (µg/kg)**	**Positive (%)**	**Median (µg/kg)**	**Maximum (µg/kg)**
*Alternaria* metabolites	Alternariol	80	10.4	37.7	93	53.6	449	100	116	305
	Alternariol-methylether	95	0.5	5.2	100	21.6	177	100	0.8	2.5
	Tenuazonic acid	15	59	247	89	21	82	21	18	35
	Altertoxin-I	95	2.7	5.6	96	7.3	36.1	96	4.5	15.7
	Tentoxin	25	0.2	0.4	93	1.3	3.6	0	-	-
	Pyrenophorol	0	-	-	93	29.4	108	0	-	-
Ergot alkaloids	Chanoclavine	40	0.1	0.2	0	-	-	18	0.1	0.9
	Ergometrine/-metrinine	10	0.6	0.6	0	-	-	11	0.6	4.9
	Ergocristine/-cristinine	25	20.2	68.3	0	-	-	4	44.2	44.2
	Ergocornine/-corninine	12	2.5	10.3	4	0.2	0.2	21	4.2	55.0
	α-Ergocryptine/-cryptinine	15	25.5	58.7	4	0.3	0.3	21	8.6	78.9
*Penicillium* and *Aspergillus* metabolites	Sterigmatocystin	15	1.0	1.2	57	2.1	20.1	7	1.0	1.0
	Mycophenolic acid	10	31.6	56.2	25	8.7	13.5	36	12.8	166.7
	Averufin	35	7.3	25.6	82	32.0	168	11	10.4	72.0
	Cytochalasin E	40	4.1	16.1	71	9.1	48.6	55	5.6	38.2
	Asterric acid	60	6.7	28.6	69	9.7	39.7	54	5.3	12.6
Other fungal metabolites	Curvularin	60	1.8	41.1	79	2.5	10.7	71	2.4	29.4
	3-Nitropropionic acid	15	3.0	5.6	11	2.0	9.4	36	4.1	22.8
	Emodin	100	15.6	75.3	100	22.3	111	100	5.4	71.5

**Figure 2:**
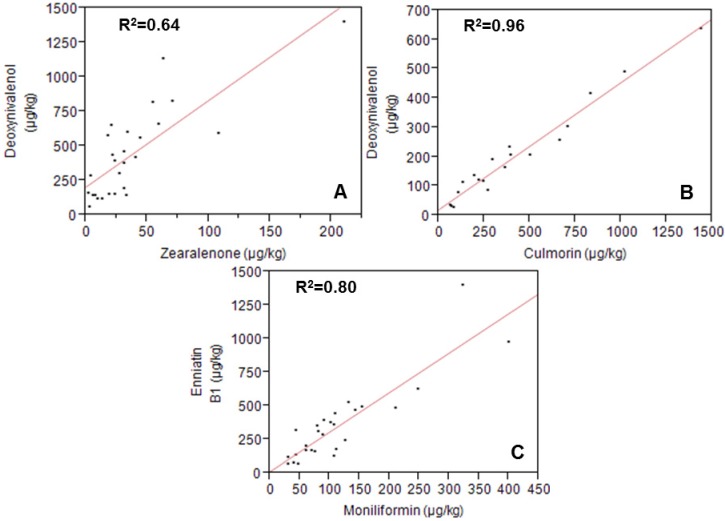
Scatter plots including squared correlation coefficients visualising linear correlations of selected fungal metabolites: correlation between ZEN and DON in wheat (*R*^2^=0.49 after exclusion of the highest concentration) (**A**), correlation between culmorin and DON in barley (**B**), and correlation between MON and ENN B1 in wheat (**C**).

The contamination of grain samples with the mycoestrogen zearalenone (ZEN) was usually low in our previous survey programmes [2]. Also this study showed that the contamination of wheat with ZEN (median of positive samples 27.5 µg/kg) is substantially lower compared to other parts of the world [16]. However, the recent pilot study showed that Norwegian oats and barley may be substantially contaminated with this compound (Table 1, Figure 1). The contamination of oats in this study (maximum concentration 1670 µg/kg) is comparable to what was found in a recent study from Sweden (2297 µg/kg) although the prevalence was significantly higher in the Norwegian samples [17]. Interestingly, the ZEN concentrations were not especially well correlated to the DON concentrations, and the correlation between the two mycotoxins was strongest in wheat (Figure 2). Furthermore, the relative concentration of β-zearalenol and ZEN-sulphate together was about 10% of that of ZEN (Table 1). The plant metabolite ZEN-14-glucoside was not detected in the samples. However, the few existing reports on the occurrence of plant metabolites of mycotoxins (“masked mycotoxins”) show that ZEN-14-glucoside in particular is a plant metabolite that needs attention in both future analytical surveys and toxicological assessments, as it can occur in higher concentrations than ZEN itself [18]. The relatively high concentration of ZEN might again be at least partly explained by an increasing occurrence of *F. graminearum* combined with extremely wet weather conditions during the summer of 2011. In fact, many places of Southern Norway experienced their wettest August and September ever in 2011 [1]. Relatively high ratios of ZEN-to-DON in UK wheat from 2004 have been suggested to be a result of a delayed, wet harvest [19].

It has previously been shown that culmorin compounds are often co-occurring with type-B trichothecenes, which could be expected considering that both *F. graminearum* and *F. culmorum* produce these compounds [20]. Also, in this pilot study, culmorin was detected in most samples, and was strongly correlated to DON production with R^2^ coefficients of 0.96 in barley and 0.92 in oats and wheat (Figure 2). Culmorin concentrations were, likewise, positively correlated to ZEN concentrations in oats (*R*^2^ = 0.89), but not in the samples from other grain species. ENNs were detected in all samples, which is in accordance with earlier reports on their ubiquitous occurrence in grain from Northern Europe (Table 1) [21]. Their concentrations, however, were generally lower than in earlier studies [22,23]. The occurrence of ENNs is certainly a result of the common infection of Norwegian grain with *F. avenaceum* [9]. Consequently, ENN concentrations were positively correlated to other metabolites that are likely to originate from the same fungus, e.g., MON (strongest correlation in wheat) (Figure 2). Another metabolite, produced by *F. avenaceum*, is the sphinganine analogue 2-AOD-3-ol [24]. Occurrence data for this compound were so far practically missing. Several barley and wheat samples contained high concentrations of this compound, while none of the oat samples were positive (Table 1, Figure 1). The concentrations were high enough to allow verification of the compound from daughter ion scanning of the protonated molecular ions.

The analysis method included some more *Fusarium* metabolites that were not detected in the samples. These were T-2 triol, fusarenon-X, fusaric acid and the fumonisins. The absence of the latter is in accordance with the absence of fumonisin-producing *Fusarium* spp. in Norway.

### 2.3. Metabolites Related to *Alternaria*

The occurrence of alternariol, alternariol-methylether and altertoxin-I was high in the analysed samples (80%–100%, Table 1). Indeed, the observed occurrence of these compounds was significantly higher than what was reported in a recent EFSA opinion on *Alternaria* toxins, where the overall occurrence in feed and agricultural commodities in Europe was reported between 6% for alternariol-methylether and 73% for altenuene. Altenuene was not detected in the present study. Alternariol was the *Alternaria* toxin of highest prevalence in the present study (80%–100%). Furthermore, oats and wheat contained significantly more alternariol (Student’s *t*, *p* < 0.05) than barley, while oats contained significantly more alternariol-methylether than wheat and barley (Student’s *t*, *p* < 0.0001). Maximum levels of alternariol were comparable to what has been reported in the literature [25].

Data on the occurrence of tenuazonic acid in grain is scarce. However, the few reports demonstrate a relatively high prevalence of this compound in grain and grain-based products. For example, Swedish oats have been shown to be contaminated with over 4000 µg/kg [26]. The concentration levels in this pilot study were significantly lower, where the maximum concentration of tenuazonic acid (247 µg/kg) was found in a barley sample (Table 1, Figure 1). Although the prevalence of tenuazonic acid was significantly higher in oats compared to barley and wheat, concentrations were generally low and below 100 µg/kg.

A recent study reported the suppression of toxin production (by more than 95%) in *Alternaria tenuissima* when the fungus was co-cultivated with *F. culmorum* or *F. graminearum* [27]. We thus plotted alternariol against trichothecene concentrations, but could not detect any correlation. The latter may in part be due to the fact that an unknown number of the samples were collective samples, *i.e.*, were from lots representing a mixture of grain from several fields, which might influence the correlation analysis.

### 2.4. Ergot Alkaloids

The major source of these tryptophan-derived alkaloids in Europe is the parasitic fungus *Claviceps purpurea* [28]. The biosynthetic end products and most important ergot alkaloids in terms of occurrence and pharmacological activity are the ergopeptines. Even though this study revealed the presence of several ergopeptine analogues in the barley and wheat samples, concentrations were generally low (Table 1).

### 2.5. Metabolites Related to Penicillium and Aspergillus

A relatively high number of oats samples (57%) contained sterigmatocystin, a metabolite that is structurally closely related to the aflatoxins. Interestingly, the occurrence and concentrations of this metabolite were significantly lower in barley and wheat. The toxic effects of sterigmatocystin are much the same as for afltatoxin B1. Consequently, the observed concentrations of 20 µg/kg must be regarded as significant (Table 1). Averufin is an anthraquinone compound that is likely to be involved in aflatoxin biosynthesis and appears to be primarily produced by *Aspergillus versicolor* [29]. This compound was found in most of the oats samples (82%) with a maximum concentration of 168 µg/kg. Another *Aspergillus* spp. metabolite, cytochalasin E, is an epoxide-containing member of the cytochalasin family [30]. This metabolite occurred in more than 50% of the samples in concentrations of <100 µg/kg (Table 1). Moreover, asterric acid is a metabolite of both *Aspergillus* and *Penicillium* spp. and was detected in lower µg/kg amounts in more than 50% of the samples. Mycophenolic acid is an immunosuppressive compound derived from several *Penicillium* spp. [31]. This compound was detected in a number of samples, and at concentrations of >100 µg/kg in two wheat samples (Table 1).

As most *Aspergillus* and *Penicillium* species primarily infect grain and other commodities during storage, it is no surprise that most of the metabolites that are related to species within these genera were not detected in the samples. In particular, we could not detect aflatoxins or ochratoxin A in the samples.

### 2.6. Toxicological Considerations

Only a few of the mycotoxins detected in the grain samples are regulated. Several of the oats samples (five out of 28) exceeded the recommended maximum EU levels for DON of 1750 µg/kg in unprocessed oats, and one wheat sample exceeded the maximum level of 1250 µg/kg in unprocessed wheat for food production given in EU Commission Regulation (EC) 1881/2006. The DON concentration was lower than the regulated maximum limit in all samples of barley. The regulated maximum concentration for ZEN in unprocessed cereals (other than maize) for food production in the EU is set to 100 µg/kg. Two barley samples, 12 oats samples and two wheat samples exceeded this maximum limit. None of the samples exceeded the guidance levels for feed production according to EU Commission Recommendation 2006/576/EC for DON and ZEN, or the indicative levels for T-2 and HT-2 toxin in cereal and cereal products according to Commission Recommendation 2013/165/EU. 

It is well-known that naturally *Fusarium* infected grain is more toxic than single *Fusarium* toxins or defined mixtures of toxins. Furthermore, there is a large variation in the dose-response relationships when pigs are given DON-containing naturally infected grain, precluding the establishment of safe levels in animal feed [32]. This indicates that, in addition to DON, other components in the infected grain contribute to the toxic effects. The results in this survey demonstrate the presence of a large number of fungal metabolites in all grain samples, emphasizing the need to focus on exposure to mixtures of fungal metabolites in future toxicological studies. Recently, natural derivatives of certain mycotoxins and mycotoxin conjugates from plant metabolism (so-called “masked mycotoxins”) have come into focus [16,33]. The grain samples included in this screening contained concentrations of 3-acetyl-DON in the range 0%–28% of free DON, but no 15-acetyl-DON. Although the acetylated forms of DON are currently included in the tolerable daily intake set by JECFA [34], this is currently not reflected in the maximum limits in the EU, where the limits apply to DON only. Moreover, the major plant metabolite of DON is its 3-glucoside, which was present in all samples, generally in the range 4%–60% of the concentrations of the unconjugated form. Two barley samples contained 91% and 153% of the glucosylated DON conjugate relative to the unconjugated compound. A recent study of rats indicated that the bioavailability of this form of DON could be low [35]. On the other hand, it has also been reported that certain strains of intestinal bacteria are able to deconjugate the glucoside and release unconjugated DON, while other strains lacked this ability [36]. Thus, the bioavailability of these conjugated forms may vary between individuals, depending on the composition of their intestinal bacterial community. The occurrence of other, unknown forms of DON has been indicated in several studies. For example, acid hydrolysis of a plant based matrix reportedly increased the DON concentrations up to 70% [37]. Clearly, more information is needed about the fate and toxicological significance of masked mycotoxins in humans and sensitive domestic animal species.

ENNs were present in rather high concentrations in all grain samples. Unfortunately, even though many *in vitro* studies investigating the toxicity of ENNs have been published, their significance *in vivo* remains unknown. The compounds have recently been demonstrated to interfere with lysosomes [38] and immunological responses *in vitro* [39], and a potential effect of ENNs alone or in combination with other mycotoxins affecting the same parameters cannot be excluded.

Furthermore, the risk related to ZEN exposure in Norway has recently been found to be low for both human and animal health [2]. However, the maximum levels of ZEN in unprocessed oats and barley in the samples analysed in this study are well above the No Observed Adverse Effect Level (NOAEL) for pigs identified in the Norwegian risk assessment [2]. The low median concentrations indicate that the levels in processed feed will be low due to the mixing during feed production. The high maximum concentrations, however, indicate that ZEN may occasionally reach effect concentrations in farms producing their own feed. Moreover, the concentrations of zearalenol and ZEN-sulphate were low, indicating that the contribution from these analogues could be expected to be low. Other reports of high levels of ZEN metabolites, particularly ZEN-14-glucoside, exceeding the concentrations of ZEN itself, indicate that the levels of ZEN analogues in grain need further attention [18].

Tenuazonic acid was present in a significant proportion of the oats samples (89%), but less in barley (15%) and wheat (21%). The levels were relatively low and within the range reported from a 10 year survey in Northern Germany [40]. Tenuazonic acid is, like trichothecenes, an inhibitor of the protein synthesis, and interactions of this toxin with the more frequently reported trichothecenes is not unlikely. Another immunosuppressant, mycophenolic acid, was likewise detected in several of the grain samples, but the concentrations appear to be low compared to therapeutic doses.

The occurrence of sterigmatocystin was surprisingly high, with 57% positive samples in oats, and a median concentration of 2.2 µg/kg in positive samples. Sterigmatocystin has been shown to be genotoxic *in vitro* comparable to the aflatoxins, but seems to be significantly less hepatocarcinogenic [41]. Since a specific maximum limit for this toxin is lacking, the levels should be assessed with reference to the maximum limit of 2.0 µg/kg for aflatoxin B_1_. Nine samples (all oats) exceeded this level. Significantly, this is the first report on the occurrence of sterigmatocystin in Norway. These findings demonstrate the need for more information on occurrence and toxicity of this metabolite. 

The *Alternaria* metabolites alternariol and alternariol methyl ether were also frequently detected in the samples. These metabolites are also known to have a genotoxic potential [25,42,43,44]. *In vivo* genotoxicity data for these compounds are lacking, but the relatively widespread occurrence implies that such studies are needed. The available data indicate that the genotoxicity has indirect mechanisms, and consequently that a threshold for the toxic effect may be assumed.

The grain samples also contain a range of other fungal metabolites for which very little toxicological information is available. The potential contributions from these toxins to the overall toxicity of the naturally infected grains remain unknown.

## 3. Experimental Section

### 3.1. Chemicals and Reagents

Methanol and acetonitrile (both LC gradient grade) were purchased from J.T. Baker (Deventer, The Netherlands), ammonium acetate (MS grade) and glacial acetic acid (p.a.) were obtained from Sigma-Aldrich (Vienna, Austria). Water was purified successively by reverse osmosis and a Milli-Q plus system from Millipore (Molsheim, France). Reference standards were either gifts from collaborating research groups or purchased from the following sources: Romer Labs (Tulln, Austria), Sigma-Aldrich (Vienna, Austria), Iris Biotech GmbH (Marktredwitz, Germany), Enzo Life Sciences (Lausen, Switzerland), Fermentek Biotechnology (Jerusalem, Israel), Bioaustralis Fine Chemicals (Smithfield, Australia), BioVioticaNaturstoffe GmbH (Dransfeld, Germany), Alfarma (Prague, Czech Republic) and LGC Promochem GmbH (Wesel, Germany) [5,6,7].

### 3.2. Samples

**Figure 3:**
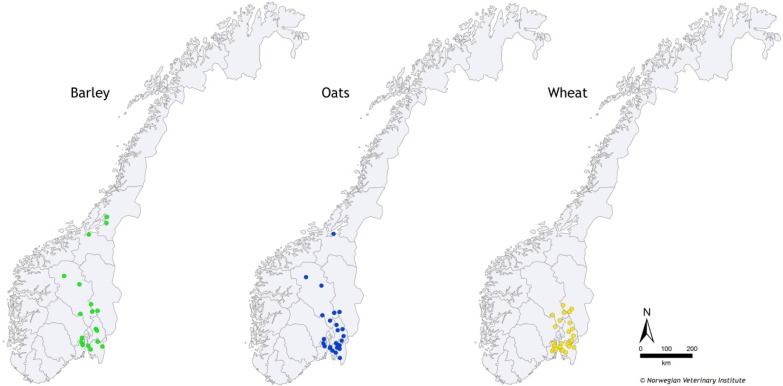
Location of sampling sites.

The 76 cereal grain samples (20 barley, 28 oats, 28 wheat) analysed in this study were collected by the National Food and Feed Authority as part of their yearly survey program at grain mills throughout the grain-producing areas in Southern Norway in 2011 (Figure 3). From each grain lot, subsamples of at least 4 kg were taken using an automatic sampler at the mills delivery site. The number of subsamples is a function of the grain lot size and is prescribed in the Sampling Directive of the National Food and Feed Authority. For example, grain lots up to 2.5 t require at least seven subsamples, and lots between 2.6 and 5 t require at least 10 subsamples [45]. The subsamples were combined to give a collective sample, which was mixed thoroughly before three new subsamples (each 500 g) were taken. A single grain lot may contain grain harvested from several farmers’ fields. Even though the location of the mills is known, the exact origin of each grain sample is unknown as grain lots are sometimes transported over larger distances within Norway depending on the mill’s capacity. Thus, a grain sample from a given mill does not necessarily reflect the contamination of local fields. Individual samples were stored at −26 °C upon arrival at the Norwegian Veterinary Institute and until further processing prior to analysis.

### 3.3. Analysis

Grain samples (500 g each) were grind using a Retsch RM 200 mill (Retsch GmbH, Haan, Germany) and aliquots (5 g) weighed into 50-mL centrifuge tubes (Greiner Bio-One GmbH, Frickenhausen, Germany). The samples were extracted using a mixture of acetonitrile/water/acetic acid 79:20:1 (*v*/*v*/*v*) by orbital shaking for two hours. After sedimentation, aliquots were transferred to chromatography vials and diluted (1:1) with acetonitrile/water/acetic acid 20:79:1 (*v*/*v*/*v*). A multi-mycotoxin LC-MS/MS method has been applied to directly analyse the diluted extracts without further pretreatment. Detection and quantification was performed using a QTRAP 5500 LC-MS/MS System (AB SCIEX, Foster City, CA, USA) equipped with a Turbo Ion Spray electrospray ionization (ESI) source and a 1290 Series HPLC System (Agilent Technologies, Inc., Santa Clara, CA, USA). Chromatographic separation was performed at 25 °C on a 150 × 4.6 mm i.d., 5 μm, Gemini C_18_ column (Phenomenex, Torrance, CA, USA) and a mobile phase consisting of methanol/water/acetic acid 10:89:1 (*v*/*v*/*v*, containing 5 mM ammonium acetate; eluent A) and 97:2:1 (*v*/*v*/*v*, containing 5 mM ammonium acetate; eluent B). After initial isocratic elution for 2 min with 100% eluent A, the proportion of eluent B was increased linearly to 50% within 3 min and to 100% within 9 min, followed by isocratic elution of the column for 4 min with 100% eluent B and 2.5 min re-equilibration of the column with 100% eluent A pumped at a flow rate of 1 mL/min. ESI-MS/MS was performed in the scheduled multiple reaction monitoring (sMRM) mode both in positive and negative polarities in two separate chromatographic runs per sample by scanning two transitions per metabolite. Detailed instrumental parameters have been reported before and can be found in the literature. 

## 4. Conclusions

The objective of this pilot study was to identify the most relevant groups of fungal metabolites present in Norwegian cereal grain. The analysed samples were from a “worst-case” season with weather conditions favouring fungal infection. The data give reason to further survey a range of fungal metabolites in addition to the most often analysed type-A and -B trichothecenes, such as zearalenone, 2-AOD-3-ol, sterigmatocystin and alternariol. Moreover, the combined effects of trichothecenes with several other fungal metabolites need to be investigated in the near future.
